# William H. Atkinson, M.D., D.D.S.

**Published:** 1891-05

**Authors:** 


### WILLIAM H. ATKINSON, M.D., D.D.S.

The profession throughout the country will feel, in a positive degree, a sense of loneliness that this remarkable man has passed from his continued life of activity to be known no more among his fellows. So prominent and so pronounced has that life been, so intensely active for the good of the profession he early adopted and loved, that it is difficult to realize that the dental science of the future will never more feel his invigorating spirit.

His forceful character has left an impress on dentistry such as is given to few men to accomplish in a generation. His peculiarities were many and oftentimes obtrusive in their character, but these will be forgotten, and when the silent roll of names is called of those who laid the foundation and helped rear the superstructure of dentistry, his will not be the least.

How almost pathetic are the closing remarks of his on the subject of "Work," made before the Odontological Society of New York, February 17!—"Personally speaking, I feel that I do not know the lapse of time, or anything as to the consequences that in my best mood invoked my most enthusiastic efforts in doing the work. But that is progress. *It is made for me.*"

His later days were days of sorrow, and if progress be for him, and for all men, we hope that the shadow that fell on his declining years may prove but the dark background to the brilliancy which is yet to be.

